# New approach methodologies to enhance human health risk assessment of immunotoxic properties of chemicals — a PARC (Partnership for the Assessment of Risk from Chemicals) project

**DOI:** 10.3389/ftox.2024.1339104

**Published:** 2024-04-09

**Authors:** Igor Snapkow, Nicola M. Smith, Emma Arnesdotter, Karsten Beekmann, Etienne B. Blanc, Albert Braeuning, Emanuela Corsini, Marija Sollner Dolenc, Loes P. M. Duivenvoorde, Gunnar Sundstøl Eriksen, Nina Franko, Valentina Galbiati, Johanna M. Gostner, Nathalie Grova, Arno C. Gutleb, Rita Hargitai, Aafke W. F. Janssen, Solveig A. Krapf, Birgitte Lindeman, Katalin Lumniczky, Ambra Maddalon, Steen Mollerup, Lucia Parráková, Arkadiusz Pierzchalski, Raymond H. H. Pieters, Maria J. Silva, Anita Solhaug, Yvonne C. M. Staal, Anne Straumfors, Tünde Szatmári, Jonathan D. Turner, Rob J. Vandebriel, Ana Claudia Zenclussen, Robert Barouki

**Affiliations:** ^1^ Department of Chemical Toxicology, Norwegian Institute of Public Health, Oslo, Norway; ^2^ Luxembourg Institute of Science and Technology (LIST), Belvaux, Luxembourg; ^3^ Wageningen Food Safety Research (WFSR), Part of Wageningen University and Research, Wageningen, Netherlands; ^4^ T3S, INSERM UMR-S 1124, Université Paris Cité, Paris, France; ^5^ Department of Food Safety, German Federal Institute for Risk Assessment, Berlin, Germany; ^6^ Department of Pharmacological and Biomolecular Sciences “Rodolfo Paoletti”, Université degli Studi di Milano, Milan, Italy; ^7^ Faculty of Pharmacy, University of Ljubljana, Ljubljana, Slovenia; ^8^ Norwegian Veterinary Institute, Oslo, Norway; ^9^ Biochemical Immunotoxicology Group, Institute of Medical Biochemistry, Medical University of Innsbruck, Innsbruck, Austria; ^10^ Immune Endocrine Epigenetics Research Group, Department of Infection and Immunity, Luxembourg Institute of Health, Esch-sur-Alzette, Luxembourg; ^11^ Unit of Radiation Medicine, Department of Radiobiology and Radiohygiene, National Centre for Public Health and Pharmacy, Budapest, Hungary; ^12^ Section for Occupational Toxicology, National Institute of Occupational Health, Oslo, Norway; ^13^ Helmholtz Centre for Environmental Research, Leipzig, Germany; ^14^ Innovative Testing in Life Sciences and Chemistry, Research Center for Healthy and Sustainable Living, University of Applied Sciences, Utrecht, Netherlands; ^15^ IRAS-Toxicology, Population Health Sciences, Faculty of Veterinary Sciences, Utrecht University, Utrecht, Netherlands; ^16^ Department of Human Genetics, National Institute of Health Dr. Ricardo Jorge, Lisbon, Portugal; ^17^ Centre for Health Protection, National Institute of Public Health and the Environment, Bilthoven, Netherlands

**Keywords:** PARC, new approach methodologies, NAMs, immunotoxicology, immunosuppression, regulatory toxicology, chemical toxicology

## Abstract

As a complex system governing and interconnecting numerous functions within the human body, the immune system is unsurprisingly susceptible to the impact of toxic chemicals. Toxicants can influence the immune system through a multitude of mechanisms, resulting in immunosuppression, hypersensitivity, increased risk of autoimmune diseases and cancer development. At present, the regulatory assessment of the immunotoxicity of chemicals relies heavily on rodent models and a limited number of Organisation for Economic Co-operation and Development (OECD) test guidelines, which only capture a fraction of potential toxic properties. Due to this limitation, various authorities, including the World Health Organization and the European Food Safety Authority have highlighted the need for the development of novel approaches without the use of animals for immunotoxicity testing of chemicals. In this paper, we present a concise overview of ongoing efforts dedicated to developing and standardizing methodologies for a comprehensive characterization of the immunotoxic effects of chemicals, which are performed under the EU-funded Partnership for the Assessment of Risk from Chemicals (PARC).

## 1 Introduction

The immune system is an immensely complex hierarchical network of cells, tissues, and organs, governed by a multitude of intricately regulated mechanisms. Beyond its primary role in protecting the body against pathogens and in responding to host-originated danger signals, the immune system plays a crucial role in a large number of biological processes including metabolism (Chapman and Hangbo, 2022), neurodevelopment and central nervous system functioning (Morimoto and Nakajima, 2019; Matejuk, Vandenbark, and Offner, 2021), pregnancy (Abu-Raya et al., 2020), as well as digestion and nutrient absorption (Zhou et al., 2020; Wiertsema et al., 2021).

Given these multifaceted properties, it is not surprising that the wide range of chemicals encountered in human life can interact with the immune system and have the potential to modify its functions. Substances capable of inducing immune dysregulation are referred to as immunotoxicants. Immunotoxicants can exert their effects through various mechanisms, including immunosuppression, immunostimulation, sensitization, the development of autoimmunity, and direct toxic effects on the cells and components of the immune system (Rooney et al., 2012). In 2022, a committee of eighteen experts identified ten key characteristics of immunotoxicants, aiming to improve the understanding of their various modes of action (Germolec et al., 2022).

While estimating the exact number of potential immunotoxic substances is challenging, a variety of immunotoxicants have been identified, including chemicals, drugs, infectious agents, pollutants, and more. At present, the list of chemicals with documented immunotoxic properties consists of per- and polyfluoroalkyl substances (PFASs), polycyclic aromatic hydrocarbons (PAHs), chlorinated solvents, pesticides, and others (Veraldi et al., 2006; Naidenko et al., 2021).

Historically, the evaluation of substances for immunotoxicity has been primarily performed using animal models, with established assays such as the T-cell-dependent antibody response (TDAR) and local lymph node assays (LLNA) (Anderson, Siegel, and Meade, 2011; Lebrec et al., 2014). However, the paradigm shift towards animal-free toxicity testing, along with the emergence of novel human-relevant *in vitro* and *in silico* testing and prediction methods, provide a tremendous opportunity to establish and standardize robust, reliable, and comprehensive new approach methodologies (NAMs) for assessing the immunotoxicological properties of various substances. Despite the highlighted demand for such novel approaches by the World Health Organization (WHO)/International Programme on Chemical Safety in 2012 (https://apps.who.int/iris/handle/10665/330098), a scientific consensus and widely accepted standards for immunotoxicity testing of chemicals in the post-animal-testing era are yet to be established. Consequently, only a limited number of *in vitro* immunotoxicity assessment methods have achieved the status of OECD test guidelines. These include TG 444A (IL-2 Luc assay evaluating the immunotoxic effects of chemicals on T-cells), TG 442D (ARE-Nrf2 luciferase test method assessing keratinocytes activation), and TG 442E (a series of tests to determine dendritic cell activation in the context of skin sensitization) (“OECD Guidelines for the Testing of Chemicals, Section 4: Health Effects, 2023”).

Hence, within the European Union (EU)-funded Partnership for the Assessment of Risk from Chemicals (PARC) (https://www.eu-parc.eu/) (Marx-Stoelting et al., 2023), the present project aims to develop and harmonize a set of methods for immunotoxicity testing, with the ultimate goal of integrating them into risk assessment and regulatory frameworks.

In this publication, we intend to provide a description of the wide array of methodologies used by consortium partners within the PARC project. The work has been grouped and described according to the respective focus areas: immunosuppression, respiratory sensitization, and various immunotoxicity pathways, as well as the characterization and identification of novel immunotoxic substances.

## 2 Immunosuppression

Immunosuppression is characterized by a reduction in the functioning of the immune system or a decrease in the quantity of its components (Hussain and Khan, 2022). Immunosuppression can be induced by either an immediate impairment of immune cells or by the stimulation of immunosuppressive mediators production, as well as the alteration of regulatory cell populations (Biologic Markers in Immunotoxicology, 1992; Huaux, 2018). Multiple chemical agents, including pesticides and other organic compounds, metals, and specific immunosuppressive medicines, have been demonstrated to exhibit immunosuppressive properties (Bou Zerdan et al., 2021). However, there exist no widely accepted chemical-agnostic *in vitro* tests specifically designed for assessing the immunosuppressive properties of chemicals (Wang et al., 2023). Therefore, there is a growing demand for developing NAMs for evaluating immunosuppression.

### 2.1 Institut national de la santé et de la recherche médicale (INSERM, France)—Immunosuppression under the antigenic challenge conditions

INSERM will focus on the investigation of immunosuppression using experimental models of vaccination response. The initial approach involves using a mouse model to monitor the response to an influenza vaccine in the presence or absence of various chemicals, including toxins and PFASs. To be able to see the effects in mice, concentrations around the lowest observed adverse effect level (LOAEL) will be used (Peden-Adams et al., 2008). Comprehensive cellular (histopathological), molecular (cytokine release) and omics (transcriptomics and targeted proteomics) analyses of animal material (mainly spleen and primary peripheral blood mononuclear cells (PBMCs)) will be conducted. The results of this study will provide a foundation for the development of *in vitro* tests. In parallel, the impact of these chemicals on primary PBMCs, whether activated or not by antigens (e.g., recall antigens) will be monitored using similar methods. Point of departure (POD) doses will be determined by PBBK modeling using publicly available biomonitoring values. Additionally, extracellular vesicles will be characterized. Ultimately, the effects on THP-1 and Jurkat cell lines, representing monocytes and T cells will be analysed and compared to the results from the previous models in order to consider these cell lines for a NAM. A systems toxicology integration of the data will be conducted with other tasks from PARC to identify common gene expression modules across the different experimental settings.

### 2.2 Norwegian Institute of Public Health (NIPH, Norway)—Assessing immunosuppression using single-cell immune profiling

NIPH will perform a comprehensive analysis of the immunosuppressive properties of PFAS and PFASs mixtures using human primary PBMCs. Initially, a systematic assessment of the cytotoxicity of the test substances will be performed to determine suitable working concentrations. Subsequently, in-depth immune profiling utilizing mass cytometry in combination with cutting-edge unsupervised data analysis methods will enable the characterization of potential immunotoxic effects at the single-cell level. The approach taken will be similar to Nygaard, et al., 2021 to explore expression of functional markers and changes to cell subsets (Nygaard et al., 2021). Additionally, the changes in cytokine levels upon exposure will be quantified using a multiplex Luminex assay. The combined data will allow to identify novel immune-related outcomes associated with PFASs exposure and will serve as valuable information for the further development of NAMs.

### 2.3 Wageningen Food Safety Research (WFSR, the Netherlands)—Transcriptomic characterization of immunosuppression

WFSR will study the effects of various test substances on existing models for immunotoxicity, such as primary immune cells and cell lines using transcriptomics, flow cytometry, and functional endpoints, covering likely key events for many immunotoxicity pathways. The work will focus on the development of a generic approach for mechanistic research on immunotoxicity. Available animal-free methodologies will be evaluated, assessing which immunological pathways the existing methods address, and selected for testing. Primary immune cells (PBMCs and/or subsets), as well as cell lines, will be treated with test substances (model immunotoxicants, mycotoxins, and priority chemicals such as PFASs) and analyzed using transcriptomics (RNA sequencing) and flow cytometry. Functional endpoints to be assessed include cytokine and antibody production.

### 2.4 Luxembourg Institute of Health (LIH, Luxembourg)—NAMs for natural killer immunotoxicity

Natural killer cells (NK) are the founding members of the innate lymphoid cell (ILC) family that are both cytotoxic and have antibody-dependent cellular cytotoxicity. LIH will study the consequences of early-life exposure to persistent organic pollutants, especially bisphenol A (BPA) analogs, on NK function, and the long-term health consequences. Our project will address the role of NK in immunosuppression based on previous human studies highlighting that these cells are durably altered in adulthood in individuals who have been exposed to environmental pollutants early in life compared with respective free living control subjects.

*In vitro*, NK cells derived from Human CD34^+^ Hematopoietic Stem Cells will be used to investigate how BPA analogs trigger immunosuppression or induce immunotoxicity, through an unbiased screening tool for flow cytometry data visualization, viSNE. LIH will specifically address the NK immune cell population immunophenotyping and its functionality (cytotoxicity and degranulation response assessment). An *in vivo* study, based on in-house established rat models of immuno-developmental toxicity will be performed on a selection of BPA analogs inducing NK immunosuppression to (i) validate the suitability of the *in vitro* model at modelling an early life adversity and (ii) to investigate by which mechanisms the immune system becomes impaired after early life exposure to these BPA analogs. The shift in the senescent state of NK cells will also be targeted.

### 2.5 University of Ljubljana (ULFFA, Slovenia)—*In vitro* substitution for whole blood cytokine release assay and investigation of glucocorticoid receptor role in lymphotoxicity

The Whole Blood Cytokine Release Assay (WBCRA) is used to assess immunosuppression by treating whole blood from a donor with lipopolysaccharide (LPS) or Staphylococcal enterotoxin B (SEB) and measuring the cytokines released (Langezaal et al., 2002; Corsini and Roggen, 2017). However, this method requires the collection of *ex vivo* blood samples and is therefore subject to inter-individual variation. Furthermore, a lack of standardization of this method has been reported in the literature (e.g., LPS concentration, timing of activation, cytokines measured, reporting of results), making it difficult to draw conclusions and compare results between laboratories (Fullerton et al., 2016). ULFFA will develop an *in vitro* model for WBCRA using cell lines that avoids the need for *ex vivo* material, is standardized in execution and provides reliable results.

The coculture will be prepared using THP-1 and Jurkat cell lines, representing monocytes and T cells. So far, the studies using these cell lines have shown them to be a promising tool for evaluation of immunosuppression. However, the existing studies have rather been focused on cytokine gene expression in each of these lines rather than the cytokine release in their coculture (Kimura et al., 2014; Zhao et al., 2019; Kimura et al., 2021). A range of activators will be investigated to assess their effects on cytokine release from each cell type and the co-culture. Following the application of known immunosuppressants belonging to different mechanisms of action, the changes in secreted cytokines will be investigated. The immunosuppressants will be applied in nanomolar and micromolar concentrations, therefore resembling *in vivo* concentrations. A panel of cytokines that change independently of the immunosuppressive mechanism will be selected as markers for immunosuppression. In the more advanced stages of the project, the lymphoblastoid cell line LCL (Markovič et al., 2015) will be added to the co-culture to bring the model closer to whole blood. Once the model is in place, we will test whether endocrine disruptors such as bisphenols and PFAS act as immunosuppressants.

Lymphotoxicity represents the second tier of the immunosuppression evaluation. The activation of T lymphocytes is mediated via glucocorticoid receptor (GR) and results in IL-2 secretion (Northrop, Crabtree, and Mattila, 1992). However, commonly used *in vitro* models for T lymphocytes are Jurkat T cells that have a mutation in GR and in this respect do not reflect the full function of T lymphocytes (Vacca et al., 1990; Riml et al., 2004). Therefore, we will also use a GR-GAL4 Luciferase Reporter Jurkat Cell Line to investigate whether T-cell inhibition is mediated via the glucocorticoid receptor or not. Other mechanisms underlying immunosuppression will be examined on THP-1 and Jurkat cell lines to exploit the effects on signaling pathways with the aim of novel biomarker identification.

## 3 Respiratory sensitization

It is widely accepted that certain low-molecular-weight (LMW) chemicals are able to create allergic sensitization of the respiratory tract resulting in hypersensitivity reactions with symptoms such as asthma and rhinitis (Cochrane et al., 2015). Occupational asthma represents about 15% of all adult asthma cases (Vincent et al., 2017). The contribution of LMW chemical exposure to disease development is likely to be underestimated due to diagnostic challenges. Inhalation is the most common exposure route for the induction of respiratory sensitization, however, skin exposure may also be an effective route (Pauluhn, Woolhiser, and Bloemen, 2005). An induction phase of immunological priming is followed by an elicitation phase triggered by the second exposure to the chemical allergen, resulting in inflammation and symptoms of a hypersensitivity reaction (Cochrane et al., 2015).

Due to the need for accurate and reliable test methods for the assessment of respiratory sensitization potential of chemicals, various testing methods are being explored. However, there are currently neither *in vivo* nor *in silico* or *in vitro* assays available that are universally accepted and validated for this purpose. It is a great challenge to develop NAMs that mimic the crucial steps known so far of the highly complex *in vivo* pathogenesis of respiratory sensitization. A single assay may not be sufficient to complete this task, but a combination of suitable assays could be used in an integrated testing strategy (Jowsey et al., 2006; Üzmezoğlu, 2021).

### 3.1 Medical University of Innsbruck (MUI, Austria)—NAMs for respiratory sensitization using 3D epithelial and immune cell co-cultures

MUI has developed a new method to assess the effect of long-term volatile chemical exposure on human lung epithelial cell models grown as air-liquid interface (ALI) cultures in a dose-dependent manner. The exposure technology is based on a previously developed incubator platform that generates a stable atmosphere containing defined concentrations of volatile compounds (Gostner et al., 2016). Exposure of ALI cultured epithelial and immune cells in co-cultures allows the evaluation of the effects of the test substances on the interactions of these cell types, as relevant biological processes involved in respiratory immunotoxicity and sensitization can be mimicked. Cytotoxicity and pathway focused readouts will be combined with expression data to identify relevant targets.

### 3.2 National Centre for Public Health and Pharmacy (NPHC, Hungary)—NAMs for the identification of respiratory sensitizers focusing on lung epithelial and dendritic cells

NPHC will explore the effects of low-molecular-weight chemical respiratory sensitizers (such as chloramine-T and piperazine) on human airway epithelial cells (BEAS-2B) and dendritic cells (DC) using a variety of cellular, biochemical and omics methods. Chemical concentrations will be determined based on cell viability assay results (da Silva et al., 2023).

Extracellular vesicles (EVs) are membrane-coated nanovesicles, actively secreted by cells (Szatmári et al., 2019). EVs contain DNA, mRNA, microRNA, lipids and proteins that are potential biomarkers of various diseases. EVs are taken up by target cells, transferring information from one cell to another, thereby influencing their behavior. It has been described that EVs in the lung play an important role in eliciting airway inflammation and allergic immune responses (Fujita et al., 2014; Zhang et al., 2021), and analyzing the EV cargo of airway epithelial cells may be used to identify substances with respiratory sensitizing potential.

NPHC aims to develop a NAM involving dendritic cells and EVs for chemical risk assessment. EVs will be isolated from the supernatant of airway epithelial cells treated with relevant chemicals. The DC activating capacity of EVs will be investigated and compared to DC activation induced by sensitized airway epithelial cells. We hypothesize that EVs and their content might be used to predict respiratory sensitization, therefore, they might represent possible NAMs. We will also conduct *in vivo* experiments to validate our *in vitro* NAM model by using EVs isolated from the bronchoalveolar lavage fluid (BALF) of sensitized mice.

### 3.3 National Institute for Public Health and the Environment (RIVM, the Netherlands)—NAMs for the identification of respiratory sensitizers using air-liquid interface models

RIVM will study the effects of exposure of the Calu-3 human bronchial epithelial cell ALI model (Braakhuis et al., 2020) to a dose range of the respiratory sensitizers: chloramine-T and piperazine. Parameters are viability, barrier function, and cytokine/chemokine production. For one of the substances, the analysis will be complemented with a primary bronchial epithelial cell model.

### 3.4 Luxembourg Institute of Science and Technology (LIST, Luxembourg)—NAMs for the identification of respiratory sensitizers

LIST will use the 3D *in vitro* model ALIsens^®^ representing the alveolar barrier (Chary et al., 2019) originally developed in-house to identify respiratory sensitizers. ALIsens^®^ is based on an older model developed to identify respiratory irritants (S. G. Klein et al., 2013; 2017; Fizeșan et al., 2018; Fizeșan et al., 2019) and consists of 4 cell lines (A549, alveolar epithelial cells; THP-1 derived macrophages (PMA differentiated); THP-1 native, dendritic cells; EA.hy 926, endothelial cells) grown at the ALI in hanging inserts (Lacroix et al., 2018; Burla et al., 2023).The two chosen substances chloramine-T and piperazine will be assessed. Parameters measured will include cell viability, surface marker expression (CD54, CD86, TSLPr), cytokine/chemokine production (including IL-6, MIP-3α, MCP-1, GM-CSF, TSLP, IL-7) and potentially also the expression of a set of defined genes (CD86, CD80, CD8a, G-CSF and others).

## 4 Inflammatory effects

As chronic inflammation is recognized as an integral part of immunotoxicity (Kanterman, Sade-Feldman, and Baniyash, 2012), the PARC project aims to enhance methods to evaluate the proinflammatory properties of chemicals.

### 4.1 Institute for Risk Assessment Sciences (IRAS, the Netherlands)—*In vitro* models for lung and liver inflammation induced by chemicals

IRAS will study the effects of BPA analogs and toxins using established NAMs which reflect hub key events (KEs) that have been linked to several adverse outcome pathways (AOPs) of inflammatory diseases such as lung and liver fibrosis (Villeneuve et al., 2018).

The three hub KE are designated “Tissue resident cell activation”, “Increased proinflammatory mediators” and “Leukocyte recruitment and activation”. These KE have also been linked to AOP for skin sensitization and food allergy and focus mainly on initiating inflammation, realizing that inflammation is heavily controlled by a range of regulatory mechanisms. IRAS will build on an inflamed intestinal model that uses co-cultures of adipocytes, intestinal epithelial and immune cells (DC and T cells). This model relates to the metabolic effects of endocrine disrupting chemicals (EDCs) on immune responses initiated by tissue-resident cells. The focus of read-out parameters will be on cell stress (KE1), formation of mucus and other proinflammatory mediators (e.g., cytokines/chemokines) (KE2), and macrophage/DC/T cell activation (KE3). This research will assess whether these models can be useful as NAMs in IATA development.

## 5 Hazard identification and hazard characterization

In the PARC project, beside the development of NAMs for immunotoxicity which are not targeting specific substances, other partners have focused on particular data-poor substances such as BPA alternatives and mycotoxins. These PARC priority substances have been extensively reviewed by PARC partners in separate publications (Kodila, Franko, and Sollner Dolenc, 2023; Louro et al., 2024).

### 5.1 Mycotoxins (*Alternaria* toxins and enniatins)

#### 5.1.1 National Institute of Occupational Health (STAMI, Norway)—Immunomodulating effects of *Alternaria* toxins

STAMI will test the immunomodulating potential of *Alternaria* mycotoxins (alternariol, alternariol monomethyl ether, altenuene, tenuazonic acid, tentoxin, altertoxin-I).

The innate immune response is crucial for early defense against infections. Certain mycotoxins inhibit the lipopolysaccharide (LPS) triggered induction of the Nuclear Factor-kB (NF-kB) pathway (Del Favero et al., 2020). Dysregulation in the NF-kB pathway, especially unappropriated activation can lead to host susceptibility to infections and disease-causing organisms (Bąska and Norbury, 2022). We will explore the potential immunomodulating effect of the *Alternaria* mycotoxins (alternariol, alternariol monomethyl ether, altenuene, tenuazonic acid, tentoxin, altertoxin-I) on NF-kB activation via innate immune receptors: toll-like receptors (TLR) and dectin receptor signalling, using HEK-BlueTM reporter cell assays for hTLR2, hTLR4, hDectin-1a and hDectin-1b (Invivogen). The reporter cells will be exposed to mycotoxins in combination with natural activators of these receptors (LPS or LTA). This system has been suggested to be a useful tool for studying the potential of microbial components in organic dust to engage the immune system (Afanou et al., 2023; Schmeisser et al., 2023).

Previous studies indicate that physiological relevance increases in line with the complexity of models (Lacroix et al., 2018), and that advanced 3D models, which are closer to tissue and organ structure, can be more reliable for *in vitro* toxicity testing in human hazard assessment (Camassa et al., 2022). To mimic occupational inhalational, exposure the biologically relevant *in vitro* human 3D mucociliary lung tissue model (EpiAirwayTM, Mattek) (Silva et al., 2023) will be used for pulmonary toxicity assessment of the *Alternaria* mycotoxins. The model will be complemented with the seeding of differentiated THP-1 cells on the apical side of the epithelium. EpiAirwayTM trans-well inserts will be exposed to mycotoxins in the ALI system, which allows for exposures to more realistic concentrations of airborne particles compared to submerged cultures (Braakhuis et al., 2020). The co-cultures will be exposed to single and mixtures of toxins (with or without co-exposure to LPS). After exposure cells will be collected for transcriptomics and pathway analysis with a particular focus on pulmonary immunotoxicity will be carried out. The results may add to the development of a battery of NAMs needed for carrying out NGRA of *Alternaria* toxins.

#### 5.1.2 Norwegian Veterinary Institute (NVI, Norway)—Studying immune effects of mycotoxins in gastrointestinal models

NVI will study the effects of enniatins and *Alternaria* mycotoxins on a commercially available human gastrointestinal model based on primary cells (EpiIntestinal ™, MATTEK). The selected mycotoxins are widely distributed in food and feed and the gastrointestinal tract is one of the first potential targets in the body. The cell model allows studies of effects on cytokines production and barrier function as well as absorption and biotransformation and intestinal morphology.

The cells will be exposed to the toxins alone or in combination with IL-1b or LPS. This will allow studies of immune inhibition as well as immunostimulation. The effects on the production of multiple cytokines will be measured using multiplex methods and the effects on the barrier will be measured by trans-epithelial electric resistance. In addition, the model allows studies of absorption and biotransformation in the gut epithelium. All data will be compared with available information from animal experiments and more traditional human cell line models to validate the relevance of the model for human risk assessment.

#### 5.1.3 Norwegian Institute of Public Health (NIPH, Norway)—Immunosuppressive actions of mycotoxins on PBMCs

NIPH aims to study the potential immunomodulatory properties of emerging mycotoxins. This research focuses on six *Alternaria* toxins and five enniatins. The project’s experimental setup involves isolating PBMCs from both healthy male and female donors, to account for any sex effects (Sankaran-Walters et al., 2013; S. L; Klein and Flanagan, 2016; Abdullah et al., 2012). These PBMCs will be exposed to *Alternaria* toxins and enniatins for 24 and 48 h.

Firstly, cell viability assays will be used to determine the appropriate dose range, with a particular emphasis on avoiding cytotoxic effects. Additionally, a Luminex multiplex assay will be used to quantify cytokine levels, providing insights into immune cell functional responses when exposed to these mycotoxins. Lastly, to provide deeper insight into immune modulation by the mycotoxins, changes in various immune cell populations and their activation status as well as functional markers will be investigated using mass cytometry, described in section 2.2 by NIPH. Together, these steps will contribute to filling data gaps on the immunotoxic potential of mycotoxins.

#### 5.1.4 German Federal Institute for Risk Assessment (BfR, Germany)—*In vivo* repeated-dose study of enniatin B1 toxicity

In addition to the various *in vitro*- and cell-based approaches delineated above, an OECD TG 408-compliant 90-day repeated-dose oral toxicity study with enniatin B1 in rats will be performed, with the aim to close data gaps regarding this substance, and to facilitate the risk assessment of enniatins. The study will first be using classic endpoints related to histopathology and clinical chemistry. In addition, tissues from the study will be utilized for NAM-based analyses. Samples from different organs will be subjected to whole transcriptome characterization, to obtain mechanistic insight into the toxicological consequences of enniatin B1 exposure, including effects on immune-related functions. More specifically directed towards immune parameters, PBMCs and spleen homogenates will be used for immunoprofiling with flow cytometry or mass cytometry. Investigation of *in vivo* material will increase our mechanistic knowledge on enniatin toxicity and comparison to *in vitro* NAM-based findings will increase confidence in the validity of *in vitro* approaches and NAMS in the field of immunotoxicity.

### 5.2 Bisphenol A analogs

#### 5.2.1 University of Milan (UMIL, Italy)—Effects of BPA analogs on primary T- and B-cells

UMIL will address the effects of BPA analogs on T cell differentiation and immunoglobulin production. Literature evidence shows the ability of BPA to interact with T cells, and mainly with T helper 17. We will use PBMCs purified from buffy coats obtained from healthy donors of both sexes. PBMC will be exposed to BA for 24 h and subsequently stimulated with anti-CD3/anti-CD28 for 4 days to induce T cell activation, differentiation and expansion. After 5 days in total, cells will be harvested, and T helper cell differentiation will be assessed through the detection of intracellular cytokines or cell surface markers (by flow cytometry) and evaluation of the cytokine release (by ELISA). CD4^+^ cells will be analyzed for the expression of IFN-γ, IL-4, IL-17A to assess T helper 1, 2, and 17 populations, respectively. Regulatory T cells, CD45^+^CD4^+^ CD127^-^ cells, will be analyzed based on CD25 and FoxP3 expression and for the absence of CD127. In parallel, the release of IFN-γ, IL-4, IL-17A, and TGF-β will be assessed in cell-free supernatants. Another important class of lymphocytes is represented by B cells, whose main role is antibody production, which can be targeted by BPA analogs. We will investigate the effect of PBMCs exposed to BPA analogs for 24 h *in vitro* with subsequent stimulation with ODN2006 and rh-IL-2 for 6 days on B cells. At the end of the treatment, total IgG and IgM production will be measured in cell-free supernatants using ELISA. In addition, as positive reference control of immunosuppression, cyclosporin A and rapamycin will be used for T cell and B cell assays, respectively.

#### 5.2.2 National Institute of Health Dr. Ricardo Jorge (INSA, Portugal)—Using co-cultures of epithelial cells with fibroblasts and macrophages to investigate the immunomodulatory effects of BPA analogs

INSA will study the effects of BPA analogs (and their mixtures) using cellular and molecular approaches. Co-cultures of polarized epithelial cells from the intestine or lung with fibroblasts and undifferentiated macrophages M0 (THP-1, a widely used model of macrophages) will be used to explore the impact of chemicals on the release of cytokines and other immune modulators (e.g., ROS, NO and PGE2), as well as changes in macrophage differentiation (M1 and M2 markers).

#### 5.2.3 Helmholtz Centre for Environmental Research (UFZ, Germany)—Creating flow cytometry test panels to identify immune cell populations affected by chemicals

Researchers at the UFZ have established a flow cytometry-based test battery for chemicals testing on T cell subsets, MAIT cells, basophils, NK and B cells by selecting unique cell type-specific stimulus and surface and intracellular activation markers (Krause et al., 2023; Maddalon et al., 2023; Pierzchalski et al., 2023; Pierzchalski et al., 2024). These tests are designed to evaluate the immunosuppressive and immunostimulatory effects in a short time. UFZ will further develop and standardize their assays by determining which immune cell types are affected the most upon incubation of human PBMCs with BPA analogs and PFASs. In a later phase of the project, the interaction between treated immune cells/placenta 3D cultures will be studied *in vitro*. This will help to assess immune cells as mediators of toxicity towards placenta and therefore contribute to the refinement of reproductive toxicology test systems.

## 6 Mechanistic constructs

### 6.1 Adverse Outcome Pathway (AOP) development (coordinated by NIPH, Norway and MUI, Austria)

As demonstrated from the large variety of projects by numerous PARC partners above, toxicologists are generating an increased volume in data by both pathway focused and high throughput omics techniques, thus expanding the existing information volume on relevant toxic effects exerted by chemicals as well as on underlying mechanisms.

This increasing amount of data and publications pose a challenge for their timely use in chemical risk assessment. Therefore, the concept of AOPs which integrate information along a series of events finally leading to an adverse outcome, considering the different levels of biological organization, is an important tool for the assessment of causality and human relevance. Importantly, AOPs support the use of NAMs to predict adverse health outcomes and hence AOPs are being developed in the same priority areas as the NAMs described above.

There is a recognised need for AOPs in the area of immunotoxicity, as these are underrepresented in the AOP wiki and needed to support regulatory decision making. During the first years of the PARC project there is a concerted effort to focus on the (further) development of AOPs in the areas of immunosuppression, respiratory sensitization and inappropriate immune enhancement (possibly leading to inflammatory diseases). Focus areas for immunosuppression are the development of existing AOP 14 (https://aopwiki.org/aops/14), related to glucocorticoid receptor activation and the inclusion of NK cell inhibition as a KE to include these important components of the innate immune system. In addition, there is work underway to further develop AOP 39, related to respiratory sensitization and to expand on the existing Molecular Initiating Events (MIEs) and KEs in the AOP wiki for sensitizer identification.

Chronic, low-grade inflammation may increase the risk of several non-communicable diseases. However, similar inflammatory processes operate in different adverse outcomes and these need to be better represented as hub KEs in several AOP framework (Villeneuve et al., 2018). Models representing these hub KEs may serve as NAMs to screen potential proinflammatory properties of chemicals. In PARC, we will incorporate inflammatory events into the targeted AOPs where appropriate, e.g., in the AOPs for fibrosis, sensitization and tumour development and non-genotoxic carcinogenesis in which inflammation is a driver of disease.

## 7 Concluding remarks

Establishing *in vitro* and *in silico* tests assessing immunotoxicity is among the most challenging objectives of NAMs development and animal-free science. The complexity of the immune system and the kinetic and dynamic interactions between its different components are not readily represented in simple assays. However, there are a number of recent advances that will help us make significant progress toward this objective. 1) The proposal for ten key characteristics of immunotoxicants provides the relevant biological targets that NAMs should address (Germolec et al., 2022). 2) Building AOPs in immunotoxicology will help in identifying the most relevant pathways and key events that NAMs should evaluate. This has been well illustrated in the case of skin sensitization (Clouet et al., 2019; Wang et al., 2023) and food allergy (Bilsen et al., 2017)**.** Developing new AOPs in immunotoxicology, as described above, is also being carried out in PARC and will feed into NAMs development (De Castelbajac et al., 2023). 3) The growing knowledge on the signaling pathways triggered by drugs such as immunosuppressants is extremely useful for identifying critical signaling steps, such as in the case of calcineurin signaling (Ulengin-Talkish and Cyert, 2023). 4) The remarkable increase in understanding of pathways activated by specific receptors, like the Arylhydrocarbon Receptor (Larigot et al., 2022) will also contribute to the further identification of the most relevant NAMs.

Since PARC is founded on science and policy interactions, we prioritize our projects accordingly. In order to support regulatory needs, the projects described here focus on respiratory sensitization and immunosuppression, which are two areas where robust assays are urgently required. However, other outcomes including inflammation promotion, developmental immunotoxicity and autoimmune diseases are also relevant. It is worth noting that the immune system is also involved in multiple diseases (e.g., cancer, gastrointestinal diseases, etc.) and, therefore, the NAMs developed here can have a wide range of applications for a spectrum of health conditions.

As the potential immunotoxicity is a significant concern for several chemicals prioritized in PARC Work Package 5, including BPA, its analogs (Kodila, Franko, and Sollner Dolenc, 2023) and certain mycotoxins (Schmutz, Cenk, and Marko, 2019; Kraft, Buchenauer, and Polte, 2021; De Felice, Spicer, and Caloni, 2023), these substances will be used in addressing data gaps and advancing the development of NAMs for immunotoxicity. Additionally, PFASs serve as model chemicals for several groups in PARC, given their ability to target the immune system (Holst et al., 2021; Ehrlich et al., 2023).

We expect this project to significantly contribute to the development of NAMs in immunotoxicology and to support the regulatory and research communities in this field. While the translation of immune tests into NAMs will be a gradual process, the effort is undoubtedly worthwhile.
